# Personalizing Functional Assessment in Chronic Obstructive Pulmonary Disease: A Validation Study of the Patient-Specific Functional Scale

**DOI:** 10.3390/jcm15010037

**Published:** 2025-12-20

**Authors:** Asma Alonazi, Monira I. Aldhahi, Rakan Nazer, Ali Albarrati

**Affiliations:** 1 Department of Physical Therapy and Health Rehabilitation, College of Applied Medical Sciences, Majmaah University, Al-Majmaah 11952, Saudi Arabia; a.alonazi@mu.edu.sa; 2 Department of Rehabilitation Sciences, College of Health and Rehabilitation Sciences, Princess Nourah bint Abdulrahman University, Riyadh 11671, Saudi Arabia; mialdhahi@pnu.edu.sa; 3 Cardiac Sciences Department, College of Medicine, King Saud University, Riyadh 11421, Saudi Arabia; raknazer@ksu.edu.sa; 4 Rehabilitation Sciences Department, College of Applied Medical Sciences, King Saud University, Riyadh 11433, Saudi Arabia

**Keywords:** Patient-Specific Functional Scale (PSFS), pulmonary function, chronic obstructive pulmonary disease (COPD), Duke Activity Status Index (DASI), physical activity assessment

## Abstract

**Background**: Personalized and accurate assessment of functional performance in chronic obstructive pulmonary disease (COPD) requires patient-centered tools that capture individualized activity limitations. The Patient-Specific Functional Scale (PSFS) is brief and clinically accessible, but its psychometric properties in COPD have not been fully established. This study aimed to evaluate the reliability and construct validity of the PSFS in individuals with COPD. **Methods**: A longitudinal psychometric evaluation was conducted with 70 adults diagnosed with COPD confirmed by spirometry. The PSFS was administered twice, 4–7 days apart, to examine test–retest reliability, standard error measurement (SEM), and minimal detectable change at 95% confidence interval (MDC_95%_). Construct validity was assessed through correlations between the PSFS, the Duke Activity Status Index (DASI), and the St. George’s Respiratory Questionnaire (SGRQ). Floor and ceiling effects were also evaluated. **Results**: Seventy participants (mean age 63 ± 11 years) completed the study. The PSFS demonstrated excellent test–retest reliability (ICC = 0.94; 95% CI: 0.90–0.96), and low SEM (0.16 points), and the MDC_95%_ was 0.44 points, with no floor or ceiling effects. Construct validity was supported by moderate positive correlations with DASI (r = 0.51, *p* < 0.001) and moderate negative correlations with SGRQ total scores (r = –0.41, *p* < 0.001). PSFS scores were not associated with demographic variables or COPD severity. **Conclusions**: The PSFS demonstrates strong psychometric properties in COPD, including excellent reliability and moderate construct validity. Its individualized approach, ease of administration, and ability to capture functional limitations beyond traditional clinical measures support its utility in both clinical practice and research for personalized functional assessment.

## 1. Introduction

Chronic Obstructive Pulmonary Disease (COPD) represents a long-term respiratory disorder characterized by airflow obstruction and chronic inflammatory responses in the airways and lungs [1]. Globally, COPD ranks among the most prevalent causes of mortality, accounting for an estimated 3.5 million deaths in 2021 and approximately 5% of all recorded fatalities [2,3]. Beyond its clinical severity, COPD imposes substantial personal and socioeconomic burdens through reduced quality of life, limitations in daily function, and escalating healthcare costs [4].

Accurate assessment of functional performance is essential for guiding clinical decision-making, monitoring disease progression, and tailoring rehabilitation interventions [5]. A wide range of instruments exists to quantify functional status in COPD, including performance-based tests and patient-reported outcome measures (PROMs) [6,7]. PROMs are particularly valuable because they capture the patient’s perception of how symptoms influence daily activities; an aspect not always reflected in physiological indices such as FEV_1_ [8].

Commonly used COPD-specific tools, such as the COPD Assessment Test (CAT) [9] and the modified Medical Research Council (mMRC) dyspnea scale [10], provide standardized assessments of symptom burden and breathlessness severity. While these measures are well established and clinically valuable [9,10], they capture general symptom severity rather than the individualized activity limitations that patients encounter in daily life. In contrast, the Patient-Specific Functional Scale (PSFS) allows individuals to identify personally meaningful activities that are most affected by their condition [11], thereby offering a complementary, patient-centered approach to evaluating functional limitations.

The PSFS is a brief, patient-centered PROM that allows individuals to identify daily activities most affected by their condition and to rate the difficulty of performing them [11]. By focusing on self-selected activities rather than a predetermined list, the PSFS provides a personalized evaluation of functional limitation. Although the PSFS has demonstrated strong reliability and validity in musculoskeletal, neurological, and cardiovascular populations, its measurement properties cannot be assumed in COPD [12,13,14,15,16]. Unlike other chronic conditions, COPD-related functional impairment is influenced by unique physiological and symptom-based factors, including fluctuating dyspnea, ventilatory inefficiency, and activity avoidance, that may affect both the activities patients select and how they rate their performance.

Despite the growing emphasis on individualized assessment in respiratory care, evidence supporting the use of the PSFS in COPD remains scarce. Validating the PSFS in this population is necessary to determine whether it accurately reflects the multidimensional functional challenges experienced by individuals with COPD and whether it performs consistently with other established measures of functional capacity and health-related quality of life.

Therefore, the purpose of this study was to evaluate the reliability and construct validity of the PSFS in individuals with COPD. By examining its test–retest reproducibility and its associations with the Duke Activity Status Index (DASI) and the St. George’s Respiratory Questionnaire (SGRQ), this study aims to determine the suitability of the PSFS as a personalized functional assessment tool within the COPD population.

## 2. Materials and Methods

### 2.1. Study Design, Setting, and Participants

This investigation employed a longitudinal cohort design to evaluate the psychometric properties of the PSFS in patients diagnosed with COPD. Ethical approval was granted by the Institutional Review Boards of King Saud University Medical City and King Fahad Medical City, Riyadh, Saudi Arabia (IRB_017E; 22-0522). All procedures adhered to the principles outlined in the Declaration of Helsinki, and informed consent was obtained from every participant prior to data collection.

A total of 70 patients with physician-confirmed COPD were recruited from the two hospitals. Eligibility was based on the Global Initiative for Chronic Obstructive Lung Disease (GOLD) diagnostic criteria [17]. Participants were aged between 30 and 80 years and were able to ambulate independently without assistive devices. Individuals using supplemental oxygen were permitted to participate. Exclusion criteria comprised those with a body mass index (BMI) ≥ 35 kg/m^2^, to reduce potential confounding from obesity-related mobility limitations, major musculoskeletal or cardiovascular disorders (e.g., advanced osteoarthritis or heart failure) that could interfere with testing, and psychiatric or cognitive impairments that might limit comprehension or safety during procedures.

#### Sample Size Calculation

The sample size for this psychometric evaluation was informed by recommendations from the Consensus-Based Standards for the Selection of Health Measurement Instruments (COSMIN) [14]. COSMIN recommendations indicate that samples of 50–100 participants typically provide sufficient precision for reliability and validity analyses and allow for the estimation of intraclass correlation coefficients (ICCs) with acceptably narrow confidence intervals. To further substantiate the adequacy of our sample, a statistical verification was performed using Walter et al.’s method for reliability studies [15]. Assuming a minimum acceptable ICC of 0.80, a target ICC of 0.90, a significance level of 0.05, and 80% statistical power, the required sample size was estimated to be 48 participants. Accordingly, the inclusion of 70 participants exceeds this threshold and offers adequate sensitivity to detect meaningful reliability coefficients. Likewise, construct validity analyses based on Pearson correlations are generally considered well powered with sample sizes greater than 50 participants. Together, these considerations support the adequacy of the sample size for the psychometric analyses conducted, while noting that larger samples may further enhance precision in future research.

### 2.2. Outcome Measures

#### 2.2.1. Sociodemographic Data

Information regarding age, sex, height, weight, BMI, and smoking history (current, former, or never-smoker) was collected. Pulmonary function test (PFT) values were also documented.

#### 2.2.2. Pulmonary Function Test

Spirometry was conducted using the Vitalograph Alpha 6000 (Vitalograph Ltd., Buckingham, UK), in accordance with American Thoracic Society (ATS) guidelines [18]. Forced Vital Capacity (FVC) and Forced Expiratory Volume in one second (FEV_1_) were recorded and expressed as percentages of predicted norms.

#### 2.2.3. Patient-Specific Functional Scale (PSFS)

The PSFS is a self-administered, patient-centered measure in which participants identify three to five daily activities hindered by their condition and rate their ability for each task on a 0-to-10 scale, where 0 represents total inability and 10 indicates full, unrestricted performance [11,14,19,20]. For this study, the PSFS was used to assess individualized activity limitations among Arabic-speaking patients with COPD. The tool is freely available and does not require licensing for clinical or research use. During the assessment, using standardized Arabic instructions, participants listed activities that they found difficult due to their respiratory symptoms [14], typically mobility tasks, household duties, or other culturally relevant activities. A total score was calculated as the mean of the activity ratings, with higher scores reflecting better functional capacity. The same activities were reassessed during follow-up to evaluate score stability and potential changes over time.

#### 2.2.4. Duke Activity Status Index (DASI)

Functional capacity was further evaluated using the DASI, a validated and freely accessible questionnaire consisting of 12 binary-response items reflecting common daily and recreational activities associated with graded metabolic equivalents (METs) [21,22]. Participants responded “yes” if they were able to perform each activity without limitation. Weighted MET values were summed to produce a total score ranging from 0 to 58.2, with higher scores representing greater functional endurance and cardiopulmonary performance [21]. The DASI total score was also used to estimate peak oxygen consumption (VO_2_ peak) using established prediction equations. The DASI has been validated in Arabic-speaking patients with COPD [22]. For consistency and clarity, the DASI was administered with standardized Arabic instructions, with assistance provided when necessary.

#### 2.2.5. St. George’s Respiratory Questionnaire

Health-related quality of life was assessed using the SGRQ, a well-established disease-specific instrument for chronic respiratory disorders. The SGRQ includes 50 items divided into three domains, Symptoms, Activity, and Impacts, collectively capturing the multidimensional burden of COPD. Domain scores and the total score are calculated on a 0–100 scale, with higher scores signifying poorer health status and greater disease impact [23]. A validated Arabic version of the SGRQ was utilized to ensure linguistic and cultural appropriateness. Participants completed the questionnaire independently or with interviewer assistance to ensure full comprehension. The SGRQ is widely distributed for non-commercial academic use, and no additional permissions or licensing arrangements were required for its implementation in this study.

#### 2.2.6. Global Rating of Change (GRC)

The GRC scale is commonly used in clinical research to evaluate patients’ perceived improvement or deterioration over time. In this study, participants rated their overall health status using an 11-point GRC scale ranging from −5 (“a very great deal worse”) to +5 (“a very great deal better”), with 0 representing no change [24]. Consistent with established conventions, participants with GRC scores between −2 and +2 were classified as clinically stable. This classification was used to support the assumption of stability required for test–retest reliability analysis of the PSFS.

### 2.3. Procedure

Prior to data collection, all participants received a standardized verbal and written explanation of the study aims, procedures, and potential risks. All assessments were conducted by trained physiotherapists experienced in cardiopulmonary rehabilitation and familiar with the administration of patient-reported outcome measures. Baseline measurements, including the PSFS, DASI, and SGRQ, were completed in a quiet, supervised clinical setting to minimize distractions and ensure comprehension. For test–retest reliability, the PSFS was administered a second time after an interval of 4–7 days. During this period, participants were instructed to maintain their usual daily routines and to report any changes in respiratory symptoms, medication use, or acute exacerbations. Clinical stability was verified through a brief structured interview at the follow-up visit, supplemented by asking participants to complete the GRC questionnaire. Only participants categorized as stable based on the GRC criteria (scores between −2 and +2) were included in the reliability analysis.

### 2.4. Data Analysis

Data analyses were performed using SPSS version 16 (IBM SPSS Statistics, Armonk, NY, USA). Demographic data (age, sex, weight, height, BMI, and smoking status) were summarized as means ± standard deviation (SD).

For cross-sectional comparisons, participants were categorized according to GOLD spirometric severity using FEV_1_ % predicted: mild (≥80%), moderate (50–79%), severe (30–49%), and very severe (<29%) [18]. Because of small group sizes at severity extremes, a secondary classification dichotomized participants into FEV_1_ ≥ 50% (mild–moderate) and FEV_1_ < 50% (severe–very severe). PSFS scores were compared across severity groups using one-way analysis of variance (ANOVA). A significance level of *p* ≤ 0.05 was considered statistically significant.

Pearson’s correlation coefficients were used to examine construct validity between PSFS and selected clinical and functional outcomes. Construct validity was measured by correlating PSFS with DASI, a measure of functional performance in patients with COPD, and the activity domain and total score of the SGRQ, with the expectation of at least moderate correlations (r > 0.40) [25].

Test–Retest Reliability was determined using the intraclass correlation coefficient (ICC_2,1_) with 95% confidence interval (CI); ICC values were interpreted as poor (<0.50), moderate (0.50–0.75), good (0.75–0.90), or excellent (>0.90) [21,25]. Measurement Error was estimated using the standard error of measurement (SEM = SD × √[1 − ICC]) and the minimal detectable change at 95% confidence (MDC_95_ = 1.96 × √2 × SEM). Bland–Altman plots were employed to evaluate agreement between repeated PSFS administrations [26]. Floor and Ceiling Effects were assessed to identify potential clustering at extreme score ranges, defined as >15% of participants achieving the minimum or maximum PSFS value [25].

## 3. Results

A total of 70 participants with clinically diagnosed COPD completed the study. Table 1 summarizes their demographic and clinical characteristics. Based on the GOLD classification, 5.7% were categorized as Stage I, 51.4% as Stage II, 38.6% as Stage III, and 4.3% as Stage IV. Most participants were male (80%) with a mean age of 63 ± 11 years, mean body-mass index of 29 ± 2.5 kg/m^2^, with a predicted FEV_1_ value of 55.98 ± 14.97%. Smoking history revealed that 55.7% of participants were former smokers, 24.3% were current smokers, and 20% had never smoked.

### 3.1. Patient-Selected Activities on the PSFS

Participants identified a range of daily activities affected by COPD. The most frequently selected tasks were stair climbing (62%), walking on level ground (58%), household activities such as cleaning or carrying objects (46%), lifting or carrying groceries (31%), and outdoor ambulation such as visiting markets or mosques (27%). These selections reflect common functional challenges associated with exertional dyspnea and reduced endurance in COPD.

### 3.2. Reliability and Error Measurements

The PSFS demonstrated excellent test–retest reliability, yielding an ICC_2,1_ = 0.94 (95% CI 0.90–0.96). The SEM was calculated as 0.16 points, and the MDC_95_ was 0.44 points. Bland–Altman analysis demonstrated good agreement between test sessions, with a mean difference of −0.08 ± 0.64 and limits of agreement ranging from −1.32 to 1.16. No systematic bias was observed. No floor or ceiling effects were observed, indicating that scores were well distributed across the functional spectrum.

### 3.3. Construct Validity

Construct validity was supported by a moderate positive correlation between PSFS and DASI (r = 0.51, *p* < 0.001) and moderate negative correlations with the SGRQ total score (r = −0.41, *p* < 0.001) and the SGRQ activity domain score (r = −0.43, *p* < 0.001), confirming that the PSFS reflects functional capacity and quality-of-life limitations among patients with COPD.

### 3.4. Associations of PSFS Scores with Demographic and Clinical Characteristics

No significant associations were found between PSFS scores and either age or BMI, indicating that patient-specific functional ratings were not influenced by demographic or anthropometric characteristics. Similarly, PSFS scores did not differ significantly between males and females, suggesting that the instrument performed consistently across sex groups without evidence of gender-related measurement bias.

PSFS scores also did not vary significantly across GOLD spirometric severity categories. One-way ANOVA showed no association between PSFS scores and GOLD stages (F (3,53) = 1.09, *p* = 0.36). When COPD severity was dichotomized at FEV_1_ ≥ 50% versus <50%, no significant differences were observed (F (1,55) = 1.95, *p* = 0.17). Together, these findings indicate that patient-perceived functional limitations captured by the PSFS are not strongly aligned with spirometric severity and may reflect dimensions of functional impairment not measurable through traditional lung function indices.

## 4. Discussion

The present study evaluated the psychometric properties of the Patient-Specific Functional Scale (PSFS) in individuals with COPD and demonstrated that the instrument exhibits excellent reliability and acceptable construct validity in this population. The high test–retest reliability observed over a one-week interval supports the stability of patient-reported functional limitations and confirms the PSFS as a suitable tool for longitudinal assessment. These findings are particularly meaningful in COPD, where functional impairment is multifactorial and not fully reflected by pulmonary function parameters alone.

The excellent reliability observed in this study (ICC = 0.94) is consistent with previous PSFS research in musculoskeletal and other chronic disease populations [25,27,28]. Importantly, our results correspond closely with findings from Albarrati et al. [29] who examined the PSFS in individuals with cardiovascular disease and likewise reported excellent test–retest reliability and moderate correlations with functional performance and disease impact measures. Their observations that the PSFS demonstrated no floor or ceiling effects and captured meaningful patient-centered limitations parallel the patterns seen in our COPD cohort, suggesting strong cross-condition consistency in the scale’s psychometric behavior. Notably, while Albarrati et al. found that the PSFS reflected functional variation in cardiovascular disease, our study showed that PSFS scores did not correspond to FEV_1_-based COPD severity, reinforcing the concept that pulmonary function alone does not adequately represent perceived activity limitation. Together, these findings indicate that the PSFS performs robustly across different chronic disease populations and that its individualized structure may be especially valuable in conditions such as COPD, where functional impairment is influenced by multidimensional factors extending beyond traditional physiological measures. 

Construct validity was supported by moderate correlations between the PSFS and both the DASI and the SGRQ. These associations indicate that the PSFS captures meaningful aspects of functional status and health-related quality of life. The DASI reflects activity-related metabolic demands, while the SGRQ evaluates symptom burden and perceived limitations; the PSFS’s alignment with these measures reinforces its relevance in assessing real-world functional capability. The moderate strength of these correlations also suggests that the PSFS provides unique information not fully captured by standardized questionnaires or physiological assessments, consistent with its patient-centered design.

The absence of significant associations between PSFS scores and demographic variables such as age, BMI, or sex aligns with previous COPD research demonstrating that functional capacity and daily physical activity are largely independent of these characteristics [25,30,31]. A previous study similarly reported that daily physical activity levels in COPD were not significantly influenced by age, sex, or spirometric severity, but were more strongly related to symptom burden and overall health status. More recently, Aldhahi and co-authors showed that walking performance is determined by a multidimensional interplay of extremity function, physical fitness, depressive symptoms, and general health status rather than by demographic factors or FEV_1_ alone [32]. These findings reinforce the notion that patient-identified functional limitations in COPD are driven primarily by disease-specific physiological mechanisms such as dyspnea severity, dynamic hyperinflation, and skeletal muscle dysfunction, rather than by demographic or anthropometric characteristics [33,34,35]. The lack of association between PSFS scores and GOLD spirometric severity categories in our cohort is also consistent with extensive evidence demonstrating that spirometry correlates poorly with functional capacity, exercise performance, and real-world activity levels [11,12,36,37]. GOLD staging captures airflow limitation but does not reflect extrapulmonary manifestations such as muscle weakness, fatigue, or behavioral avoidance, which have a substantial influence on daily functioning [4,17,32,38]. Taken together, these observations support the value of the PSFS as a patient-centered measure capable of capturing meaningful aspects of functional limitation that are not reflected in demographic profiles or airflow obstruction.

The value of individualized, patient-generated outcome measures is increasingly recognized in chronic disease management [11,12,27]. In COPD, where functional limitations vary widely across individuals with similar spirometric severity, capturing patient-prioritized activities provides clinically actionable insights that complement traditional assessments. The absence of floor and ceiling effects in this study further supports the PSFS’s discriminatory capacity across a spectrum of disease severities.

### 4.1. Study Limitations

Despite these strengths, the study has limitations. Responsiveness to clinical change was not evaluated; thus, the utility of PSFS for detecting improvement after pulmonary rehabilitation or medical interventions remains to be established. Additionally, correlations with objective activity metrics, such as accelerometry or wearable monitoring technologies, were not examined. Furthermore, although the BMI exclusion threshold was revised to ≥35 kg/m^2^ to better reflect the COPD population, this criterion may still limit sample representativeness, as individuals with severe obesity were not included. Future research should address these gaps by examining the PSFS’s responsiveness, establishing its minimal clinically important difference (MCID), and evaluating its integration with digital health tools. Larger, more inclusive studies that incorporate a broader BMI range would also help clarify the generalizability of the PSFS within comprehensive COPD assessment frameworks.

### 4.2. Clinical Implications

The PSFS demonstrated strong reliability and satisfactory construct validity, supporting its use as a complementary functional assessment tool in COPD. Its individualized structure allows patients to identify personally relevant activity limitations, facilitating patient-centered goal setting and enhancing the clinical utility of rehabilitation programs. The simple administration and minimal time burden further increase its practicality in routine care. Given the absence of floor and ceiling effects, the PSFS may be effectively applied across a broad range of COPD severities to monitor functional status and guide personalized interventions.

Moreover, the ease of administration makes it a practical tool for busy clinical settings, reducing assessment burden compared to longer performance-based tests such as the six-minute walk test (6MWT) [34]. This highlights its potential utility in routine evaluation of functional status and progress monitoring of rehabilitation interventions.

## 5. Conclusions

This study supports the PSFS as a reliable and valid tool for assessing patient-specific functional limitations in individuals with COPD. The instrument demonstrated excellent test–retest reliability, moderate associations with established functional and health-status measures, and strong discriminatory capacity without floor or ceiling effects. Owing to its patient-centered nature and ease of use, the PSFS represents a valuable addition to clinical practice and research. Its integration into routine assessment may enhance individualized care, support monitoring of disease progression, and improve evaluation of rehabilitation outcomes. Further studies are needed to examine its responsiveness and applicability in broader COPD populations.

## Figures and Tables

**Table 1:** Descriptive characteristics of the COPD participants (N = 70).

Parameters	Mean	SD	Range	
			Min	Max
Age (years)	63	11	37	88
BMI (kg/m^2^)	23.90	4.35	17.42	34.93
Smoking status, n (%)				
Non-smoker	14 (20)			
Smoker	17 (24.29)			
Ex-smoker	39 (55.71)			
FEV_1_ (L)	1.573	0.5484	0.52	2.82
FVC (L)	2.849	0.7314	1.64	4.8
FEV_1_/FVC ratio	54.73	11.32	26.8	70
FEV_1_ predicted (%)	55.98	14.97	26.3	102.8
Gender, n (%)				
Male	56 (80)			
Female	14 (20)			
GOLD stage, n (%)				
I	4 (5.71)			
II	36 (51.43)			
III	27 (38.57)			
IV	3 (4.29)			
SGRQ Total Score	49.14	24.50	4.57	93.02
SGRQ-Activity Score	56	27.57	3.27	100
Estimated VO_2_ peak *	19.07	6.85	10.98	34.83
DASI estimated METs	5.39	1.95	3.08	9.89
PSFS_total_1st	3.44	1.16	0.00	7.50
PSFS_total_2nd	3.36	1.12	0.00	7.50
DASI Total Score	21.56	15.93	2.75	58.20

Abbreviations: Patient-Specific Functional Scale (PSFS); Duke Activity Status Index (DASI); forced expiratory volume in 1 s (FEV_1_); forced vital capacity (FVC); Global Initiative for Chronic Obstructive Lung Disease (GOLD); Metabolic equivalents (METs); St. George’s Respiratory Questionnaire (SGRQ). *: Estimated VO_2_ peak was calculated indirectly using a validated prediction equation based on functional capacity measures rather than direct cardiopulmonary exercise testing (CPET).

## Data Availability

Data is unavailable due to privacy and ethical restrictions.
